# Studying the quantity component of seed dispersal effectiveness from exclosure treatments and camera trapping

**DOI:** 10.1002/ece3.4068

**Published:** 2018-05-02

**Authors:** Claudia M. Campos, Silvina Velez, María Florencia Miguel, Sofía Papú, Mónica I. Cona

**Affiliations:** ^1^ Instituto Argentino de Investigaciones de las Zonas Áridas (UNCuyo‐Gobierno de Mendoza‐CONICET) Mendoza Argentina; ^2^ Instituto Argentino de Nivología, Glaciología y Ciencias Ambientales (UNCuyo‐CONICET) Mendoza Argentina

**Keywords:** animal visits, frequency of interactions, frugivory, fruit removal, intensity of interactions, mammals, Monte, *Prosopis flexuosa*

## Abstract

The quantity component of effectiveness of seed dispersal by animals is determined by two events: fruit removal (intensity of the interaction) and animal visitation to the plant (frequency of interactions). Considering dispersal of *Prosopis flexuosa* seeds as case study*,* this work aimed at investigating the strengths and weaknesses of the two methods for assessing the quantity component of seed dispersal effectiveness: exclosures and camera traps. *Prosopis* fruits were offered for 48 hr. Exclosure treatments were performed using two types of wire‐screen cages, allowing access to ants (“closed exclosure”) and to small mammals up to 100 g (“open to small mammals”), and a treatment without exclosure (“open to all removers”). The camera trapping experiment was carried out using vertically oriented cameras placed at approximately 1.80 m height and focused on the fruits. The cameras were set in “motion detect mode,” taking series of three consecutive photographs. The exclosures largely allowed estimation of fruit removal by size‐based groups of animals, but did not provide information on species identity. In contrast, camera traps were able to identify all visitors to species level and could not only determine the number of visits by each species but also the proportion of visits, which resulted in removal of fruits. Camera trapping allowed discriminating among small mammals playing different roles, without underestimating fruit removal by scatter‐hoarding species. The quality of estimation of the quantity component of seed dispersal is remarkably better when the camera trapping method is applied. Additional information obtained, such as activity patterns of visitors, can contribute to a better understanding of the seed dispersal process.

## 1 INTRODUCTION

The seed dispersal cycle mediated by animals describes a complex succession of processes whereby fruits produced by a plant are removed by animals that disperse the seeds, some of which might germinate to seedlings and recruit to adult plants (Wang & Smith, 2002). Animal visits and removal of fruits and seeds from the source plant are the first step of seed relocation, influencing the distribution and availability of fruit for the next generation (Wang & Smith, 2002).

In the framework of seed dispersal effectiveness (Schupp, 1993; Schupp, Jordano, & Gómez, 2010, 2017), the effectiveness of frugivores as seed‐dispersing agents depends on both the quantity of dispersal, or amount of seed dispersed, and the quality of dispersal, or the probability that viable seeds are deposited at sites with high prospects of successful establishment (Schupp, 1993; Schupp et al., 2010, 2017). In this model, the quantity component of success is determined by animal visitation and fruit and seed removal (Schupp et al., 2017). Seed dispersal effectiveness can be compared with interaction strength, a concept used in food webs and in predator–prey interactions, which has recently expanded to mutualisms (Schupp et al., 2017; Vázquez, Morris, & Jordano, 2005). Thus, fruit or seed removal and animal visitation could be considered estimators of intensity and frequency of interactions, respectively. Whereas removal is measured as the number of fruits or seeds moved away from the mother plant by animals, animal visitation is defined as the number of visits by each individual that was recorded during observations at focal trees during timed observation periods (Vázquez et al., 2005).

Seed and fruit removal are considered the net result of frugivore activity and can lead to seed dispersal away from the parent plant when performed by seed‐dispersing species, instead of by seed predators (Jordano & Schupp, 2000). When fruit manipulation is performed by endozoochorous dispersers, fruit removal can be defined as the number of seeds swallowed and transported (e.g., Holbrook & Loiseller, 2009). When fruits are taken by hoarding animals to a suitable site away from their competitors and predators, fruit removal sometimes results in temporary relocation, where the fruit is consumed and seeds can be discarded, hoarded, or preyed upon (Cousens, Dytham, & Law, 2008). In earlier studies, during the 1970s and 1980s, this last kind of removal was often assumed to be synonymous with seed predation, even though the role of dispersers and ultimate seed fate were not examined (Forget & Wenny, 2005). Currently, it is known that animals that scatter‐hoard seeds in the soil, returning later to eat many but not all of them, could be considered effective seed dispersers (Price & Jenkins, 1986; Vander Wall, 2002; Vander Wall & Beck, 2012).

Forget and Wenny (2005) reviewed and examined the strengths and weaknesses of the methods used to record fruit removal and, when possible, determine the ultimate destination and fate of seeds. Research was grouped into three categories: studies using direct visual observation of animals in the wild or in captivity; studies in which seeds were attached to a fixed point using spool‐and‐line methods; and studies using marked seeds or fruits. Added to these, the use of exclusion experiments is an extended method for studying seed and fruit removal by animals. By covering seeds and fruits with wire‐mesh barriers or exclosures to selectively allow or prevent access to groups of animals based on body size, the relative importance of seed and fruit removal by animals can be inferred from the difference in their disappearance rates (e.g., Beckman & Muller‐Landau, 2007; Campos, Giannoni, Taraborelli, & Borghi, 2007; Ansley, Pinchak, & Owens, 2017).

More recently, camera trapping started being used to study seed and fruit removal, mainly by terrestrial frugivorous animals, although research is expanding into tree layers (e.g., Otani, 2001; Jayasekara, Takatsuki, Weerasinghe, & Wijesundara, 2003; Rivas‐Romero & Soto‐Shoender, 2015). Some studies have combined direct observation or experiments with feeding platforms or selective exclosures with the use of camera traps, in order to record the animal presence, recognize species, and quantify fruit removal (e.g., Yasuda, Miura, & Hussein, 2000; Beck & Terborgh, 2002; Kitamura et al., 2004; Babweteera, Savill, & Brown, 2007; Christianini & Galetti, 2007; Kitamura, Yumoto, Poonswad, Suzuki, & Wohandee, 2008; Nakashima, Lagan, & Kitayama, 2008). Applying this technology helped overcome the limitations of most available methods, such as identification of species removing fruits and visiting trees, detection of nocturnal animals and species extremely wary of human presence, quantification of removed seeds and fruits, and disturbance minimization during observation (Seufert, Linden (née Heikamp), & Fischer, 2009; Prasad, Pittet, & Sukumar, 2010).

Camera trapping can be a valuable tool to expand knowledge of the natural history, activity patterns and visitation rates of frugivorous species (Rivas‐Romero & Soto‐Shoender, 2015). Animal visitation, a measure of interaction frequency as was explained before, is partly determined by species abundance; thus, abundant animal species tend to interact more frequently than rare species (Vázquez et al., 2005). Sometimes, when the number of fruits removed is difficult to record, visitation rates are used as an estimator of fruit removal, assuming a correlation of the number of times animals visited trees and the length of their stay with the number of fruits ingested per time (Breitbach et al., 2012; Grünewald, Breitbach, & Böhning‐Gaese, 2010). Nevertheless, when seed removal is estimated from visitation rates, it is important to consider that many visitors do not remove seeds (Howe, 1986), which makes it necessary to distinguish between fruit removal events (when fruits are actually removed by an animal) and situations involving animals that simply walk past the fruiting tree without consuming fruit (mere visitors to fruiting trees) (Prasad et al., 2010). Depending on the study design and the species involved, camera trapping can allow recording the number of visits (removing fruits or not) and the number of fruits removed, both subcomponents of the quantity component of seed dispersal effectiveness.

Considering that exclosures and camera trapping are two methods currently used for investigating the quantity component of seed dispersal effectiveness, this work aimed to assess their strengths and weaknesses using removal of *Prosopis flexuosa* fruits, which are palatable to a wide range of fauna.

## 2 MATERIALS AND METHODS

### 2.1 Study area and species

This study was conducted in the Man and Biosphere Ñacuñán Reserve (34°02′S, 67°58′W, 12,800 ha; Mendoza Province, Argentina), located in the Monte biogeographic province (Cabrera, 1976; Ojeda, Campos, Gonnet, Borghi, & Roig, 1998). The climate is semi‐arid, with cold dry winters (−13 to 10°C) and warm rainy summers (20 to 42°C). Average annual rainfall is 326 mm (Ojeda et al., 1998). The mesquite woodland is the most representative community; it consists of a tree layer of *P. flexuosa* and *Geoffroea decorticans*, a shrub layer with dominance of *Larrea divaricata, L. cuneifolia* and *Condalia microphylla*,* Atriplex lampa,* and a grass layer of *Panicum urvilleanum, Pappophorum* spp. *Trichloris crinita,* and *Digitaria californica* (Ojeda et al., 1998). The reserve was established by law in 1961, with the aim of protecting the mesquite woodland from the severe logging and cattle overgrazing that the area was undergoing, and it was fenced in 1972. Since then, the reserve offers a unique situation for research because it is the only area in the Monte where grazing by domestic animals is excluded (Ojeda et al., 1998).

*Prosopis flexuosa* blooms in spring (October to December), and fruits start to ripen in summer (February). The production of *P. flexuosa* fruits shows a marked spatial and annual variability (32–100 kg/ha; Dalmasso & Anconetani, 1993). The fruit is an indehiscent pod, approximately 16 cm long, which holds 12–22 seeds. The fruit has a relatively soft exocarp and thick mesocarp, which contains the major portion of sugars, starch, and protein. Seeds (24–40 mg and 5 mm long) are within a woody endocarp, which provides physical protection, and have an impermeable coat that provides dormancy (Kingsolver, Johnson, Swier, & Teran, 1977). When ripe fruits fall, their persistence beneath tree canopies is short because animals remove most of the fruits and seeds within no more than 6 weeks after they reach the ground (Campos et al., 2007; Villagra, Marone, & Cony, 2002). It has been proposed that, in the Pleistocene, *Prosopis* species were dispersed through endozoochory by currently extinct megaherbivores (Bucher, 1987; Mehringer, 1967).

Currently, many animal species interact with fruits and seeds of *P. flexuosa*. Some ants (*Acromyrmex lobicornis, A. striatus*, and *Pheidole bergi;* Hymenoptera: Formicidae) remove fruit segments and disperse seeds along their way and around the nests (Milesi & López de Casenave, 2004; Pirk, di Pasquo, & López de Casenave, 2009). Among vertebrates, mammals are the main *P. flexuosa* fruit removers (Campos, Campos, Miguel, & Cona, 2016; Miguel, Cona, & Campos, 2017), and the only bird recorded removing fruits from the ground was *Rhea americana* (greater rhea; Struthioniformes: Rheidae) (Campos et al., 2014). Some medium‐sized mammals (weight greater than 3 kg) act as opportunistic frugivores that disperse seeds through endozoochory, such as *Dolichotis patagonum* (mara; Rodentia: Caviidae), *Lagostomus maximus* (plains viscacha; Rodentia: Chinchillidae), *Lycalopex griseus* (Argentine gray fox; Carnivora: Canidae), and the exotic *Lepus europaeus* (European hare; Lagomorpha: Leporidae) (Campos & Ojeda, 1997). Rodent species can also behave as scatter‐hoarding seed dispersers, such as *Microcavia australis* (southern cavy; Rodentia: Caviidae; weight 200–300 g; Campos et al., 2017) and as larder‐hoarding seed predators (*Graomys griseoflavus* and *Akodon dolores*) (gray leaf‐eared mouse and grass mouse; Rodentia: Cricetidae; weight less than 100 g; Giannoni et al., 2013). Also other species were recorded removing *P. flexuosa* fruits, such as *Zaedyus pichiy* (pichi; Cingulata: Chlamyphoridae), *Conepatus chinga* (Molina's hog‐nosed skunk; Carnivora: Mephitidae), *Ctenomys mendocinus* (Mendoza tuco‐tuco; Rodentia: Ctenomyidae), and *Thylamys pallidior* (pallid fat‐tailed opossum; Didelphimorphia: Didelphidae), but their roles in the seed dispersal process remain unknown (Campos et al., 2016).

### 2.2 Fruit removal experiments

#### 2.2.1 Exclosure experiment

Experiments were conducted at the end of the fruiting season in May 2014 avoiding rainy days and full moon, which may affect animal activity (Bowers, 1988). Sixty sampling stations were established under randomly chosen *P. flexuosa* trees with similar crown diameter (approximately 5 m). The minimum pairwise distance between trees was 500 m. At each sampling station, 20 *P. flexuosa* fruits containing 300 seeds in total (approximately 15 seeds per fruit), were offered for 48 hr.

Considering functional groups as sets of species showing similar effects on major ecosystem processes (Gitay & Noble, 1997), the following functional groups were defined based on previous studies (Campos & Ojeda, 1997; Campos & Velez, 2015; Campos et al., 2008, 2017; Giannoni et al., 2013): “opportunistic frugivores,” “scatter‐hoarders,” and “seed predators.” Species with as yet unknown functional roles were grouped as “others.”

**Figure 1:**
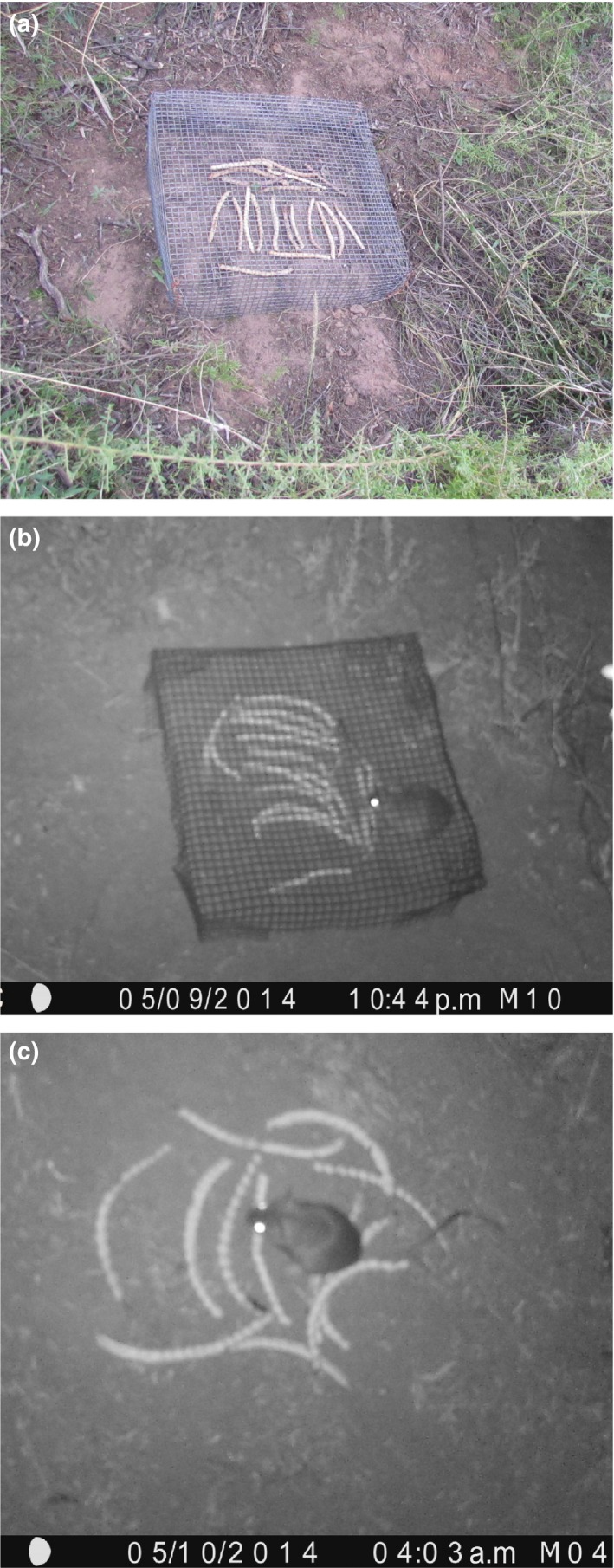
Exclosure treatments: (a) “closed exclosure,” (b) “open to small mammals,” (c) “open to all removers”

**Figure 2:**
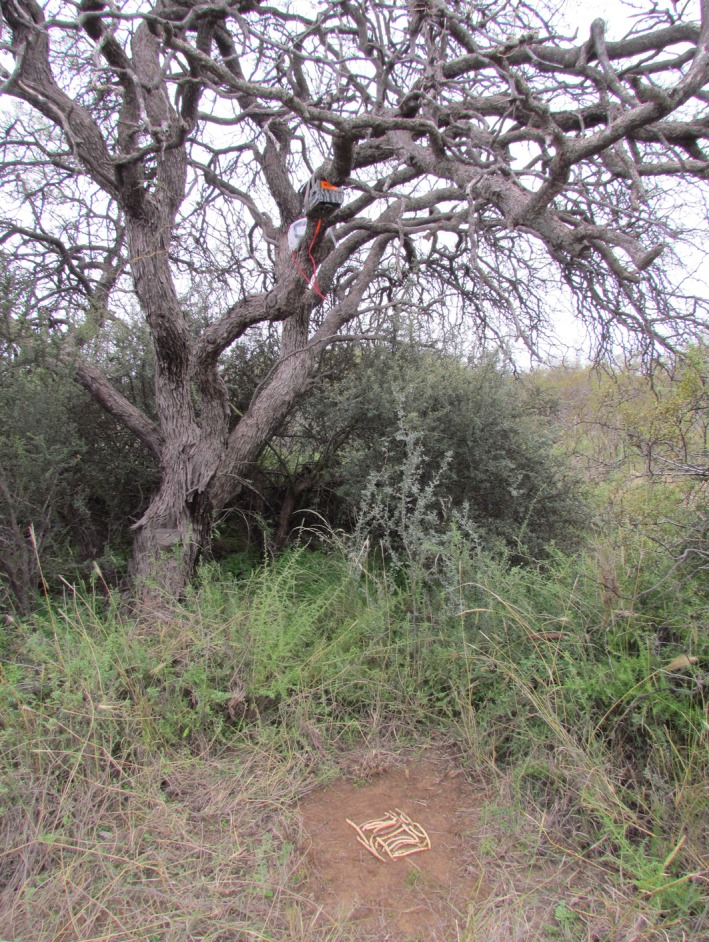
Sampling station using camera trapping. Vertically oriented cameras (Moultrie 990i) placed at 1.80 m height focused on 20 *Prosopis flexuosa* fruits

**Figure 3:**
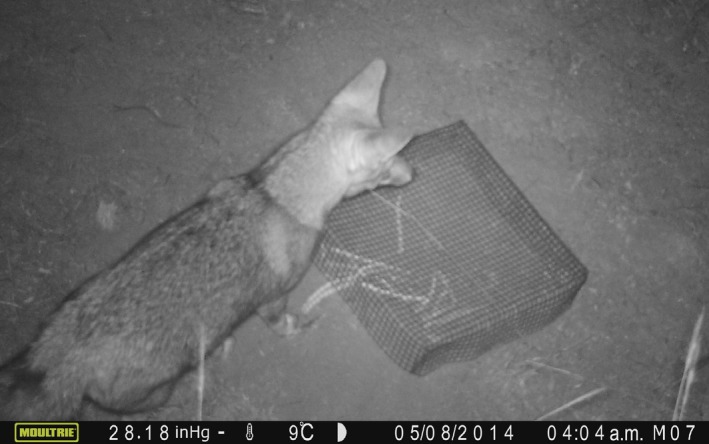
*Leucalopex griseus* lifting the exclosure and removing *Prosopis flexuosa* fruits from the “closed exclosures”

One of three treatments was randomly applied at each of 60 sampling stations, and it was assumed that exclosures effectively excluded all individuals of each desired body‐size group of animals (Campos et al., 2007; Velez, Chacoff, & Campos, 2016): (1) “closed exclosure” (using exclosure cages made of 0.75 cm × 0.75 cm wire‐screen, sized 50 cm × 50 cm × 10 cm, which prevented access of mammals but allowed access to ants (Figure 1a); (2) “open to small mammals” (using exclosure cages of similar dimensions and wire, but with four 4 cm × 6 cm openings on each side, which prevented access of mammals weighing more than 100 g (Figure 1b); (3) “open to all removers” (without exclosure, allowing access to all fauna) (Figure 1c). At the end of the second day, the number of fruits left in each treatment was recorded.

Because treatments selectively allowed or denied access to groups of animals based on body size (ants, small mammals, and vertebrates larger than 100 g), cameras were set up on trees, as is described below, in order to confirm the data recorded in every sampling station.

#### 2.2.2 Camera trapping experiment

The same 20 trees under the “open to all removers” treatment were used for the camera trapping experiment. As ants are not caught by cameras and because they remove only fruit segments and seeds, whole fruits were provided for 48 hr in order to prevent fruit removal by ant species (Milesi & López de Casenave, 2004; Pirk et al., 2009). Vertically oriented cameras (Moultrie M‐990i, Alabaster, AL, USA) were placed at approximately 1.80 m height focused on the 20 *P. flexuosa* fruits (300 seeds) provided at each of the 20 sampling stations (Figure 2). The number of fruits supplied and the survey time allowed quantifying fruit removal and identifying the vertebrate species visiting the trees (e.g. Campos et al., 2016; Miguel et al., 2017). To prevent false triggers, the vegetation was cleared in a 1 square‐m area around the fruits. Previously tested for their best setting, the cameras were set in “motion detect mode” to take three consecutive photographs once movement was detected and, with a 30‐second delay, another three photographs if the animal kept moving, and at high sensitivity to detect small mammal species (<100 g), but not ants. This setting, similar to the program proposed by Prasad et al. (2010), allowed obtaining a sequence of photographs, and the number of removed fruits was inferred by comparing the number of fruits seen in earlier and later photographs in the sequence. Thus, from individually analyzed photographs, the setting and location of the cameras allowed identifying species, counting the number of fruits removed by animals, and quantifying the number of visits with or without fruit removal (Campos et al., 2016; Miguel et al., 2017). Removed fruits were those an animal cached and moved away from the camera's coverage range. Animal species were identified by pelage color, tail and body length, and other species‐specific physical traits (Campos, Tognelli, & Ojeda, 2001; Tognelli, Campos, & Ojeda, 2001; Wilson & Reeder, 2005).

### 2.3 Data analysis

#### 2.3.1 Fruit removal according to exclosures

To determine total fruit removal and the relative contribution of different animal groups according to their access to exclosures, the data included in the analysis were obtained from the three different treatments. Fruit removal by small mammals was quantified from the “open to small mammals” treatment minus removal by ants obtained from the “closed exclosure” treatment; removal by species larger than 100 g was estimated from fruit removal in the “open to all removers” treatment minus mean fruit removal by small mammals.

The Mann–Whitney–Wilcoxon test was used to compare fruit removal by small mammals and by removers larger than 100 g.

#### 2.3.2 Fruit removal according to camera trapping

Because camera trapping allows species identification, analyses can assess differences in fruit removal among species in addition to functional groups. The number of fruits removed per tree by each animal species during visits made in a 48‐hr period was considered an estimator of the intensity of the interaction. The frequency of interactions was calculated by summing up all independent visits, with or without fruit removal, of each animal species to every focal tree. The following cases were considered independent visit events: consecutive records of individuals of different species, nonconsecutive records of individuals of the same species, or consecutive records of individuals of the same species taken more than 2 min apart.

In order to build an estimator of the quantity component of seed dispersal effectiveness and considering that subcomponents of effectiveness are multiplicative (Schupp et al., 2010, 2017), the total number of fruits removed by each species was multiplied by the total number of visits to every tree. In order to compare the quantity component of *Prosopis* fruit dispersal effectiveness among different animal species and functional groups (“opportunistic frugivores,” “scatter‐hoarders,” and “seed predators”), a zero‐inflated mixed model with a Poisson error structure (Zeileis, Kleiber, & Jackman, 2008; Zuur, Ieno, Walker, Saveliev, & Smith, 2009) was fitted, because a higher amount of zeros than expected for a Poisson distribution was detected. Trees were considered a random factor.

## 3 RESULTS

### 3.1 Seed removal according to exclosures

No seed removal was recorded from the “closed exclosure” treatment; thus, seed removal by ants was effectively prevented, as expected, owing to the short time the whole fruit was offered. The data from two exclosures were eliminated because vertebrates could reach the fruits by lifting the exclosure (*L. griseus*) or getting under it (*G. griseoflavus*), activities confirmed by cameras (Figure 1).

Results about fruit removal by small mammals and mammals larger than 100 g are presented in Table 1. According to the results obtained from the “open to small mammals” treatment, small mammals removed 66.50% of the total fruits offered. Using the mean value for fruit removal by small mammals (13.30 fruits) for correction of the data obtained from the “open to all removers” treatment, it could be estimated that animals larger than 100 g removed 19.25% of the total fruits offered (Table 1). Small mammals removed significantly more seeds than animals larger than 100 g (*W* = 318; *p* = .0011).

**Table 1:** Data obtained using exclosures and camera trapping. Animals are grouped according to the possibilities each method offers. Data include means ± *SE* of total fruits removed (intensity of interactions), total visits to trees (frequency of interactions), visits with fruit removal, and total number of trees visited by animals

Animals	Fruits removed	Total visits	Visits with fruit removal	Number of trees visited
Using exclosures				
Ants	0.00 ± 0.00			
Small mammals (up to 100 g)	13.30 ± 1.91			
Vertebrates more than 100 g	3.85 ± 0.71			
Using camera trapping				
Animal species				
*Graomys griseoflavus*	10.55 ± 1.99	11.75 ± 2.33	6.10 ± 1.31	17
*Akodon dolores*	0.05 ± 0.05	0.05 ± 0.05	0.05 ± 0.05	1
*Microcavia australis*	2.05 ± 1.24	4.20 ± 3.00	1.45 ± 4.50	5
*Lycalopex griseus*	1.4 ± 0.89	0.30 ± 0.16	0.30 ± 0.73	4
Functional groups				
Seed predators	10.60 ± 2.00	11.80 ± 2.33	6.15 ± 1.31	17
Scatter‐hoarders	2.05 ± 1.24	4.20 ± 3.00	1.45 ± 4.50	5
Opportunistic frugivores	1.4 ± 0.89	0.30 ± 0.16	0.30 ± 0.73	4
Visitors				
*Chaetophractus vellerosus*	0.00 ± 0.00	0.10 ± 0.07	0.00 ± 0.00	2
*Leopardus geoffroyi*	0.00 ± 0.00	0.05 ± 0.05	0.00 ± 0.00	1
*Asthenes* sp.	0.00 ± 0.00	0.05 ± 0.05	0.00 ± 0.00	1

**Figure 4:**
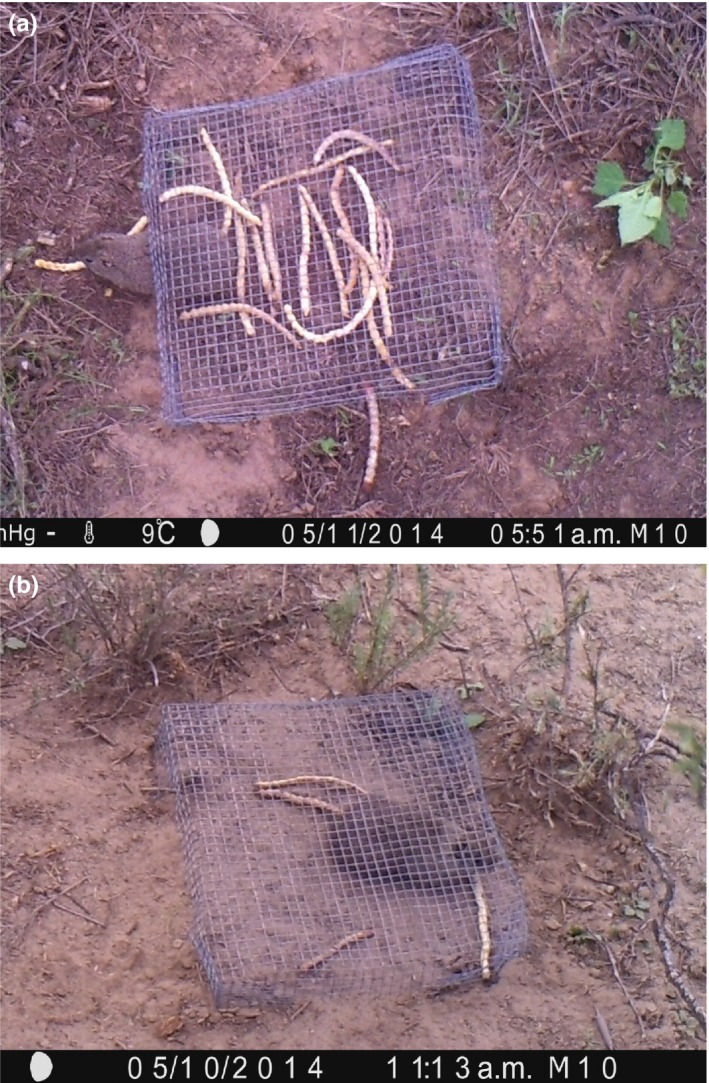
Rodent species larger than 100 g accessing “open to small mammals” exclosures. (a) *Microcavia australis*, (b) *Galea musteloides*

Using cameras to confirm the assumptions of this method regarding the groups of removers able to reach fruits in each treatment, it was found that: (1) some removers larger than ants were able to reach fruits and seeds from “closed exclosures” (Figure 3); (2) “open to small mammals” exclosures were accessed by rodents larger than 100 g (*M. australis, Galea musteloides,* and *C. mendocinus*) that were expected to remove fruits in the “open to all removers” treatment (Figure 4); (3) birds did not enter the “open to small mammals” exclosures although they approached and investigated around them.

### 3.2 Seed removal according to camera trapping

During the 40 camera‐trap nights, cameras captured a total of 1,280 photographs of animal activity. Seven species were recorded visiting trees but not all of them removed fruits (Table 1). Of the total fruits offered, 70.25% were removed only by mammals. Four species (three rodents and a fox) were recorded removing *Prosopis* fruits, and they were classified according to their functional roles: *G. griseoflavus* and *A. dolores* (seed predators), *M. australis* (scatter‐hoarding seed disperser), and *L. griseus* (opportunistic frugivorous seed disperser).

The quantity component of seed dispersal effectiveness, composed of intensity (number of removed fruits) and frequency (number of animal visits) of interactions, is higher in *G. griseoflavus* in comparison with the other removers (*z* = 343.68; *p* < .001). Nevertheless, this species is a *Prosopis* seed predator (Giannoni et al., 2013), and, because of this, the quantity component becomes higher for the seed predator role than for the other roles (*z* = 339.74; *p* < .001).

Using the date and time of each photograph, results were recorded about the daily activity of species visiting trees (Table 2). In this case, the data from photographs snapped for all exclosure treatments (“open to small mammals,” “closed exclosures,” and “open to all removers”) were considered. The main fruit removers, *G. griseoflavus* and *M. australis*, were strictly nocturnal and diurnal, respectively.

**Table 2:** Daily activity of species visiting trees in the Ñacuñán Reserve. Data from photographs snapped for all exclosure treatments (“open to small mammals,” “closed exclosures,” and “open to all removers”) were considered

Class	Family	Species	Records	Time
Mammals	Canidae	*Lycalopex griseus*	139	20:00–05:00
	Cricetidae	*Graomys griseoflavus*	3,940	19:00–08:00
	Cricetidae	*Akodon dolores*	4	19:00–08:00
	Caviidae	*Microcavia australis*	923	08:00–18:00
	Caviidae	*Galea musteloides*	21	09:00–11:00
	Dasypodidae	*Chaetophractus vellerosus*	15	11:00–16:00
	Felidae	*Leopardus geoffroyi*	30	02:00–06:00
	Mephitidae	*Conepatus chinga*	7	21:00–04:00
	Ctenomyidae	*Ctenomys mendocinus*	63	23:00–08:00
	Didelphidae	*Tylamys pallidior*	3	05:00
Birds	Furnariidae	*Asthenes*	6	10:00–11:00
	Rinocryptidae	*Rinocrypta lanceolata*	16	09:00–18:00
	Emberizidae	*Zonotrichia capensis*	9	10:00–11:00
	Mimidae	*Mimus* sp.	57	08:00–18:00

## 4 DISCUSSION

Over a long time, like in other drylands around the world, experiments using exclosures were used in the Monte to study granivory trough estimation of removal of commercial seeds by ants, birds, and small rodents (e.g., López de Casenave, Cueto, & Marone, 1998; Giannoni, Dacar, Taraborelli, & Borghi, 2001; Sassi, Tort, & Borghi, 2004). Following a similar method, sometime later, studies of fruit and seed removal from native trees, such as *P. flexuosa,* by small rodents and ants were performed (Campos et al., 2007; Velez et al., 2016), substantially improving bait‐removal experiments that only inferred fruit and seed removal by animals by observing signals of foraging activity around the trays (Villagra et al., 2002). Nevertheless, although the exclosure method provides no information about species identity, it allows obtaining data from groups of removers according to their body size, following assumptions regarding animal accessibility to exclosures. Currently, these experiments are still used but, even in a complex hierarchical exclusion design, authors recognize that they cannot be certain of a direct association between each treatment level and the fruit‐removing species targeted for that level, because each treatment level includes *Prosopis* fruit removal by the new animal species in addition to that by all other groups that previously had access to fruits (Ansley et al., 2017). In the present study case, results of fruit removal obtained using exclosures showed two main groups of removers, small mammals (<100 g) and vertebrates larger than 100 g, but each group included a wide spectrum of fruit removers where the identity of species, which have different ecological roles in seed dispersal, cannot be established.

Regarding the assumptions of the method involving exclosures, results showed that the “closed exclosures” treatment needs to be reinforced in order to prevent fruit removal by large animals able to lift the exclosures with their strength or dig to get into them. For example, exclosures could be more firmly attached to the ground and a wire‐mesh skirt could be added to prevent digging. In the case of the “open to small mammals” treatment, access by species larger than 100 g, expected to visit the “open to all removers” treatment, was not prevented. *Microcavia australis*, which is larger than 100 g, was able to access the “open to small mammals” exclosure. As this species has a different functional role in seed dispersal than small mammals <100 g, which are mainly seed predators (Giannoni et al., 2013), using the exclosures alone would have overestimated the contribution of small mammals to seed predation and underestimated seed dispersal by scatter‐hoarding mechanisms (Campos et al., 2017).

In the framework of seed dispersal effectiveness (Schupp, 1993; Schupp et al., 2010, 2017), the quality of estimation of the quantity component of seed dispersal is remarkably better when applying the camera trapping method, because it allows determining the number of fruits removed (intensity of interactions) and the number of visits to trees (frequency of interactions) by animal species. Added to this, data regarding visitors, removing fruits or not, and activity patterns of species are also available and contribute to a better understanding of the seed dispersal process. The results of this study agree with previous publications which found, also using camera trapping, that *G. griseoflavus* is the main species removing *Prosopis* fruits in the Ñacuñán Reserve. The implications of fruit removal by *G. griseoglavus* in this fenced protected area were widely discussed, as well as the differential contribution of mammal species and functional groups to fruit removal in protected areas under different management interventions (Campos et al., 2016), in grazed landscapes (Miguel et al., 2017), and at sites connecting protected and grazed areas (Tabeni, Miguel, Campos, & Cona, 2017). In this sense, it was found that opportunistic frugivores and scatter‐hoarders become important as fruit removers in grazed areas and unfenced reserves, probably due to the effect of large herbivores, including domestic animals, on habitat complexity. Recording of the data using camera trapping allowed identifying frugivorous species and discriminating among small mammals playing different roles without underestimating fruit removal by scatter‐hoarding species. Finally, it was possible to record the activity pattern of different species.

Several advantages of studying fruit removal using cameras were pointed out by Prasad et al. (2010). Remarkable among them is the capability to recognize animal species interacting with fruits and seeds, including nocturnal and hard‐to‐observe frugivores, infrequent visitors, and animals that avoid humans or are influenced by the presence of an observer, and to obtain photographs that make it clear whether or not visitors remove fruits. Also, camera trapping allows quantifying fruit and seed removal, when the cameras are suitably placed and set up according to the species involved. In this study, the vertical orientation of the cameras, the height at which they were placed, the focus on fruits, together with the setting based on series of three consecutive photographs once movement was detected, with a 30‐s delay between series, and at high sensitivity to detect small mammal species, help identify animals visiting the fruits and quantify fruit removal. In long‐term studies, when ants have more time to remove and transport fruit segments whereas camera traps are unable to detect ant activity, combining the two methods could be a good option. In this case, the use of a closed exclosure in the sampling station where cameras are placed helps correct for ant removal. Added to this, a long‐term study design should consider fruit replenishment at sampling stations, because it could turn out very difficult to quantify fruit removal by individuals using photographs if large quantities of fruits are offered only once.

Although camera trapping is a more expensive method than exclosures and thus cost becomes an important limitation to the extent of its use, this study found that camera trapping is an effective method for investigating the quantity component of seed dispersal, which allows quantifying fruit removal and identifying the vertebrates that visit the trees and remove fruits.

## CONFLICT OF INTEREST

The authors have no conflict of interest to declare.

## AUTHORS’ CONTRIBUTIONS

C.M.C. and S.V. conceived the ideas and designed methodology; C.M.C., S.V., M.F.M., and S.P. conducted fieldwork. M.I.C. performed laboratory work. C.M.C. analyzed the data with the contribution of M.I.C. and M.F.M. C.M.C. wrote the manuscript. All authors contributed critically to the drafts and gave final approval for publication.
